# Influence of PD‐1 and PD‐1L Immune Exhaustion Receptors on Immune Reconstruction in People Living With HIV

**DOI:** 10.1155/jimr/2462382

**Published:** 2025-09-30

**Authors:** Bogusz Aksak-Wąs, Karolina Skonieczna-Żydecka, Miłosz Parczewski, Rafał Hrynkiewicz, Filip Lewandowski, Karol Serwin, Kaja Mielczak, Adam Majchrzak, Franciszek Lenkiewicz, Paulina Niedźwiedzka-Rystwej, Poorani Gurumallesh

**Affiliations:** ^1^ Department of Infectious, Tropical Diseases and Acquired Immunodeficiency, Pomeranian Medical University in Szczecin, Szczecin, Poland, pum.edu.pl; ^2^ Department of Biochemical Science, Pomeranian Medical University in Szczecin, Szczecin, Poland, pum.edu.pl; ^3^ Institute of Biology, University of Szczecin, Szczecin, Poland, us.szc.pl; ^4^ Center for Experimental Immunology and Immunobiology in Infectious Diseases and Cancer, University of Szczecin, Szczecin, Poland, us.szc.pl; ^5^ Regional Center for Digital Medicine, Department of Genomics and Forensic Genetics, Faculty of Medicine, Pomeranian Medical University in Szczecin, Szczecin, Poland, pum.edu.pl

**Keywords:** CD4, CD8, HIV, immune reconstruction, PD-1

## Abstract

**Introduction:**

The progressive immunological impairment associated with human immunodeficiency virus (HIV) infection is partially mediated by the programmed cell death protein‐1 (PD‐1)/programed death‐ligand 1(PD‐L1) inhibitory pathway. This investigation aims to evaluate the influence of PD‐1 on immune reconstitution in patients undergoing antiretroviral therapy (ART), with data visualized through principal component analysis (PCA).

**Materials and Methods:**

Data from 52 ART‐treated individuals achieving viral suppression were analyzed over 12 months. CD4+, CD8+, CD19+, and PD‐1/PD‐L1 expressions were quantified via flow cytometry at baseline and after 12 months, and immune recovery was assessed at CD4+ thresholds of 500 and 800/μL and CD4+/CD8+ ratios of >0.8 and >1.0 using linear and logistic regression. PCA was applied to visualize clustering of immune recovery patterns based on PD‐1/PD‐L1 expression levels and immune cell counts, with statistical significance evaluated using ANOVA.

**Results:**

The analyzed group of 52 patients was predominantly male (65.4%; *n* = 34). PD‐1/PD‐L1 expression showed modest associations with immune recovery. Higher PD‐L1 expression on CD3+ T‐cells at baseline was associated with a reduced likelihood of recovery to CD4+>500/μL (OR: 0.79; 95%CI: 0.62–0.99; *p* = 0.04). Linear regression demonstrated that increased PD‐L1 on CD4+ T‐cells and PD‐1 on CD19+ B‐cells positively correlated with higher CD4+/CD8+ ratios at follow‐up (coefficient: 0.035 and 0.03, respectively; *p* < 0.02), while logistic regression indicated that higher PD‐1 on CD3+ T‐cells increased the odds of recovery to CD4+>500/μL (OR: 1.03; 95% CI: 1.0036–1.07); = 0.03). Notably, this weak signal may result from a general increase in the number of lymphocytes during therapy. PCA did not reveal significant clustering of immune recovery patterns.

**Conclusion:**

PD‐1 and PD‐L1 expressions on immune cells are weakly associated with immune recovery metrics in individuals undergoing ART. Further research is needed to explore their role in immune reconstitution and potential clinical applications.

## 1. Introduction

Since the introduction of highly active antiretroviral therapy (HAART), or combined antiretroviral therapy (cART), significant advancements in the therapeutic management of human immunodeficiency virus (HIV) infection have been realized, effectively transforming HIV from a terminal disease into a manageable chronic condition [1]. Despite cART’s efficacy in suppressing viral replication and extending the lifespan of individuals living with HIV, challenges remain, particularly concerning chronic immune activation and immune exhaustion [2]. Chronic HIV infection is characterized by sustained immunological activation and the overexpression of inhibitory receptors such as programed death‐1 and its ligand, programed death‐ligand 1 (PD‐L1), on various subsets of T and B lymphocytes, contributing to immune dysfunction and the persistence of HIV [3].

### 1.1. The Programmed Cell Death Protein‐1 (PD‐1)/PD‐L1 Pathway in HIV

PD‐1 is an inhibitory receptor expressed on T and B lymphocytes and monocytes, and its ligand, PD‐L1, is present on antigen‐presenting cells and various tissues. The interaction between PD‐1 and PD‐L1 attenuates T‐cell activity, a critical mechanism for maintaining immune homeostasis and preventing autoimmunity. PD‐1 overexpression on T and B cells and increased PD‐L1 expression are markers of immune exhaustion. This overexpression is associated with lymphocyte depletion, the loss of effector functions, premature apoptosis, and impaired immune response. While such phenomena occur predominantly in chronic viral infections—including hepatitis B virus (HBV), hepatitis C virus (HCV), herpes simplex virus (HSV), Epstein–Barr virus (EBV), varicella‐zoster virus (VZV), cytomegalovirus (CMV), and HIV—they are also observed in acute viral infections like SARS‐CoV‐2 [4].

In the context of chronic HIV infection, persistent antigenic stimulation results in sustained PD‐1 expression on T cells, contributing to the exhaustion of both T and B lymphocytes. Exhausted T cells exhibit diminished proliferation, reduced cytokine production, and impaired cytotoxic functions, hindering the immune system’s capacity to control HIV replication and eliminate infected cells. As such, the PD‐1/PD‐L1 pathway is pivotal in the immune exhaustion observed in chronic HIV infection [5, 6].

Research on immune cell exhaustion in HIV infection has demonstrated that elevated PD‐1 levels are associated with the compromised functionality of CD8+ T cells, characterized by a reduced capacity for cytokine and effector molecule production and impaired proliferative ability. Increased PD‐1 expression has also been correlated with higher viral loads and decreased CD4+ T‐cell counts [7, 8]. Furthermore, studies indicate that elevated PD‐1/PD‐L1 levels negatively influence HIV disease progression [7]. Significantly, HIV‐specific CD4+ T cells expressing PD‐1 are enriched during chronic HIV infection, suggesting that targeting PD‐1 with antibodies may represent a potential strategy to address the latent viral reservoir [9].

### 1.2. The Importance of Immune Reconstruction in HIV

Studies examining the natural history of HIV infection have estimated that during the initial 8 years of follow‐up, plasma viremia increases by approximately 0.04 log copies/mL per year [10]. Moreover, it is projected that, if left untreated, the disease progresses in virtually all individuals with HIV. The typical rate of CD4+ lymphocyte decline during HIV infection is about 30–40 cells/μL per year. Notably, even patients classified as elite controllers, who maintain undetectable viral loads, can experience a loss of up to 53 CD4+ lymphocytes/μL per year [10]. The progression of immunodeficiency is inhibited by effective antiretroviral therapy (ART).

Immune reconstitution represents a critical clinical endpoint in the management of HIV‐infected patients, and complete immune recovery may not occur despite optimal therapy and achievement of undetectable viral loads. Significant variability exists in the reconstitution of CD4+ T cells and the maintenance of normal cell counts, owing to factors such as age, comorbidities, and mortality rates [11]. Only a minority of individuals achieve normal CD4+ T‐cell counts in peripheral blood or lymphoid tissues. Up to 20% of patients may experience immunological failure despite suppression of HIV replication, with or without limited increases in CD4+ T‐cell counts [12–14].

Immune recovery can be assessed through various metrics. In this manuscript, we considered both the restoration of CD4+ lymphocyte counts to 500 and 800 cells/μL of blood as well as the normalization of the CD4+/CD8+ ratio. We evaluated thresholds of >0.8 and >1.0. A CD4+ lymphocyte count exceeding 500 cells/μL and a CD4+/CD8+ ratio greater than one as benchmarks derived from observations in uninfected individuals, where the CD4+/CD8+ ratio is typically above 1. A CD4+ count of 500 cells/μL is frequently regarded as a target for immune restoration, aligning with clinical categorization as per the 1993 Centers for Disease Control and Prevention (CDC) guidelines [15]. The threshold of >800 CD4+ lymphocytes/μL was selected as an indicator of more complete immune recovery, observed in a limited subset of patients, particularly those who initiate care with a CD4+ count above 500 cells/μL [16]. The attainment of 800 CD4+ lymphocytes/μL has also been associated with a reduced risk of cardiovascular mortality in other studies on immune restoration [17, 18].

### 1.3. Study Rationale and Hypotheses

Despite the efficacy of cART in suppressing HIV replication and improving patient survival, complete immune reconstitution has not been universally achieved among treated individuals [19, 20]. Persistent immune activation and immune exhaustion remain significant obstacles, even in patients who have attained viral suppression. The PD‐1/(PD‐L1) inhibitory pathway is critically involved in regulating immune responses and the development of immune exhaustion [4]. PD‐1 and PD‐L1 overexpression on T and B lymphocytes is associated with impaired immune function, including reduced proliferation, decreased cytokine production, and diminished cytotoxic activity [21–23].

Previous studies have demonstrated that elevated PD‐1 expression correlates with higher viral loads and lower CD4+ T‐cell counts, suggesting a negative impact on immune recovery [24, 25]. However, the extent to which PD‐1/PD‐L1 expression affects immune reconstitution during cART remains unclear. Understanding the relationship between PD‐1/PD‐L1 expression and immune recovery could provide valuable insights into the mechanisms underlying incomplete immune restoration and guide the development of adjunctive therapies designed to enhance immune function in HIV‐infected individuals.

We hypothesize that elevated PD‐1 and PD‐L1 expression on T and B lymphocytes is associated with impaired immune reconstitution in HIV‐infected patients undergoing effective ART. The specific hypotheses are presented below.

### 1.4. Baseline Hypothesis

Higher baseline levels of PD‐1 and PD‐L1 expression on CD4+ and CD8+ T cells, as well as CD19+ B cells, predict poorer immune recovery, as measured by smaller increases in CD4+ T‐cell counts and lower CD4+/CD8+ ratios after 12 months of cART.

### 1.5. Longitudinal Hypothesis

Changes in PD‐1 and PD‐L1 expression levels over the 12‐month treatment period are associated with immune recovery outcomes. A decrease in PD‐1/PD‐L1 expression is anticipated to correlate with better immune reconstitution, while sustained or increased expression may be linked to suboptimal recovery.

By evaluating the influence of PD‐1 and PD‐L1 expressions on immune cells and their association with immune recovery metrics—including CD4+ T‐cell counts and CD4+/CD8+ ratios—we aim to elucidate the role of the PD‐1/PD‐L1 pathway in the immune reconstitution of HIV‐infected individuals undergoing cART. The findings may contribute to the development of novel therapeutic strategies targeting immune exhaustion pathways to enhance immune restoration in this population.

## 2. Materials and Methods

Longitudinal data were collected from 52 patients followed up at the Department of Acquired Immunodeficiency, Pomeranian Medical University in Szczecin, Poland. The study protocol was approved by the Bioethical Committee of the Pomeranian Medical University (Approval number KB‐006/40/2023; date: 31.05.2023). Written informed consent was obtained from all participants, and the data were fully pseudoanonymized before being used in statistical analyses.

The entry criteria were all patients with a newly diagnosed HIV infection attending the local clinic, regardless of gender, age, HIV viral load, or CD4+ lymphocyte count. There were no exclusion criteria. Patients were assessed at the time of entry into care and after 12 months (+/−3 months). More than 90% of patients had a follow‐up in the 12th month of the study; the inertia period of +/−3 months concerned only a very few patients who did not report for testing at the scheduled time. HIV subtype was assessed at entry into care by assessing variables, such as CD4+/CD8+ immunoprofile and the percentage of PD‐1 and PD‐1 ligand receptors on CD3+CD4+, CD3+CD8+, and CD19+ lymphocytes.

Various observation endpoints were analyzed to analyze immune reconstruction. According to the CD4+ lymphocyte count thresholds of 500 and 800 cells/μL of blood was measured, as immune reconstruction, as well as the normalization of the CD4+/CD8+ ratio, evaluating thresholds of >0.8 and >1.0.

The criterion of the effective antiretroviral treatment was set for immunological restoration analyses. All patients had their first ARV treatment in the first week after admission into the outpatient clinic. The patient had to achieve undetectable viremia within 6 months in the PCR test, and the number of HIV copies in 1 mL had to be <200, which indicates the maintenance of undetectable viremia throughout the observation period.

### 2.1. Immunophenotyping

Immunophenotyping was performed using a BD FACSCanto II flow cytometer (BD Biosciences, Franklin Lakes, NJ, USA) and BD FACSDiva software version 9.0. Whole blood samples (100 µL) were incubated with monoclonal antibodies conjugated with fluorescent dyes (all from BD Biosciences), according to the manufacturer’s protocol. The antibody panel included anti‐CD3, anti‐CD4, anti‐CD8, and anti‐CD19 to distinguish major lymphocyte subsets (CD3+CD4+ and CD3+CD8+ T cells, as well as CD19+ B cells). Immune exhaustion markers were assessed through additional staining performed using anti‐PD‐1 and anti‐PD‐L1 antibodies.

After surface staining, red blood cell lysis was performed using BD FACS Lysing Solution (BD Biosciences), followed by incubation in the dark at room temperature for 15 min. The samples were then washed twice with 1000 µL of BD FACSFlow solution and centrifuged at 250 × *g* for 3 min. The final cell pellet was resuspended in 500 µL of FACSFlow and immediately subjected to cytometric analysis. Doublet discrimination was performed using FSC‐A vs. FSC‐H gating, and lymphocyte populations were defined using FSC vs. SSC plots. Subsequent gating included identification of CD3+ T cells, followed by delineation of CD4+ and CD8+ subsets, as well as CD19+ B cells. Due to sample volume limitations, fluorescence minus one (FMO) controls were not performed. Gating of PD‐1 and PD‐L1 expression was based on isotype‐informed thresholds and applied uniformly across all samples using a conservative approach.

**Figure 1:**
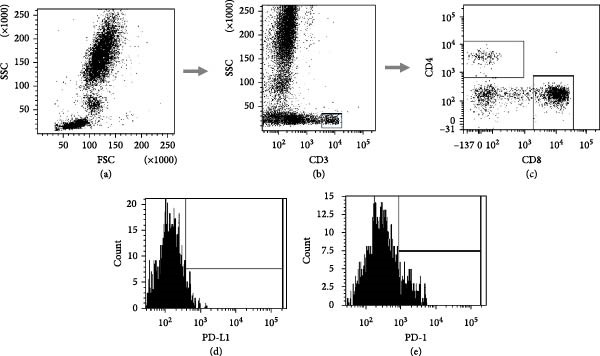
Gating strategy for immunophenotyping of T and B lymphocytes expressing PD‐1 and PD‐L1. Representative flow cytometry plots showing the gating strategy used to identify lymphocyte populations and assess expression of immune checkpoint markers. (a–c; from a): Initial gating of lymphocytes based on forward scatter (FSC) and side scatter (SSC) parameters → selection of CD3+ T cells → further discrimination of CD4+ and CD8+ T‐cell subsets. (d, e): Histograms illustrating representative fluorescence intensity distributions of PD‐L1 and PD‐1 expression on gated lymphocyte subsets.

Figure 1 depicts a representative gating strategy. For each sample, a minimum of 10,000 lymphocyte events (up to 50,000) were acquired. The percentage of PD‐1+ and PD‐L1+ cells was recorded within each gated subpopulation.

### 2.2. Statistical Analysis

This study employed a statistical approach integrating both linear and logistic regression models to evaluate factors influencing immune reconstruction in individuals over a 12‐month observation period. The analyses focused on CD4+ lymphocyte counts, CD4+/CD8+ ratios, and their deltas. The primary goal was to identify predictors of immune reconstruction at 12 months, with an emphasis on baseline and endpoint immune parameters. The analyses were done using MedCalc version 23.0.2 (Ostend, Belgium).

Principal component analysis (PCA) was conducted on the dataset to evaluate clustering patterns influenced by the categorical variable Immune reconstruction (800 and 500 cells). The quantitative variables included all columns labeled, representing a set of FC‐measured variables.

The quantitative data were standardized using scikit‐learn’s StandardScaler to ensure equal contribution from all variables. PCA was applied to extract the first two principal components (PC1 and PC2), which explain the largest variance in the dataset.

Clustering patterns were visualized in a PCA scatter plot, with confidence ellipses representing 95% certainty regions for each group. The proportion of variance explained by PC1 and PC2 was calculated to indicate how much of the dataset’s variability is captured by these components.

Additionally, ANOVA was conducted on the PC1 and PC2 scores using scipy to statistically assess whether the immune reconstruction categories significantly influence clustering patterns.

The analysis was performed using Python 3.11 and the following open‐source libraries:

1. pandas: For data manipulation and cleaning, including handling the dataset and preparing it for analysis.
2. scikit‐learn: To perform PCA and standardize the quantitative data.
3. matplotlib: To visualize the PCA results, including scatter plots and confidence ellipses.
4. scipy: To conduct ANOVA tests to evaluate the statistical significance of clustering patterns.

## 3. Results

### 3.1. Patient Characteristics

The study group consisted of 52 individuals (34 males; 65.4%), with an average age of 39 (IQR: 32–46) years. HIV subtype A6 was the most prevalent subtype (*n* = 25; 45.5%), followed by HIV subtype B (*n* = 18; 40%) and HIV subtypes G and C (*n* = 1; 2.2% each).

Median baseline CD4+ was 338.5 (IQR: 187.5–524.5) cells/μL. After 12 months of observation, the median CD4+ recovery was 596.0 (IQR: 281–774) cells/μL. The median baseline CD4+/CD8+ ratio was 0.34 (IQR: 0.15–0.91). After 12 months of observation, the median CD4+/CD8+ ratio was 0.59 (IQR: 0.35–1.08). The number of patients whose reconstructed number of CD4+ lymphocytes reached 500 cells/μL was 28 (53.8%), and the number of patients whose reconstructed number of CD4+ lymphocytes reached 800 cells/μL was 12 (23.1%). Furthermore, 20 patients (38.5%) had a reconstructed CD4+/CD8+ ratio >0.8, while 14 (26.8%) had a ratio of >1.0 was 14 (26.8%).

### 3.2. Influence of HIV Subtype on Immune Reconstruction

The most prominent HIV subtypes were A6 (*n* = 25; 45.5%) and B (*n* = 18; 40%). Patients with these two subtypes were compared in the immune reconstruction analysis. For HIV subtype A6, 12 patients (48%) reconstructed to CD4+ >500 cells/μL, nine (36%) reconstructed to >800 cells/μL, nine (36%) reconstructed to CD4+/CD8+ ratio >0.8, and seven (28%) reconstructed to CD4+/CD8+ ratio >1.0. For HIV subtype B, 10 patients (55.6%) reconstructed to CD4+ >500 cells/μL, one (5.6%) reconstructed to >800 cells/μL, five (27.8%) reconstructed to CD4+/CD8+ ratio >0.8, and three (16.7%) reconstructed to CD4+/CD8+ ratio >1.0. None of these changes reached statistical significance (*p*  > 0.5). The delta of CD4+ cells measured after 12 months of observation and baseline was 155 cells/μL (IQR: −6.0–323) for HIV subtype A6 and 204 cells/μL (IQR: 167–357) for HIV subtype B (*p* = 0.34).

### 3.3. Influence of PD‐1 and PD‐1L on Immune Reconstruction

Immune exhaustion receptors PD‐1 and PD‐1L were measured on CD3+CD4+ and CD3+CD8+ T lymphocytes, as well as globally on all CD3+ T lymphocytes and CD19+ B lymphocytes at baseline and at the 12‐month (+/−3 months) endpoint. Descriptive statistics of baseline and endpoint data regarding these variables are presented in Table S1.

To gain a better understanding of changes in immune exhaustion, we measured the delta of those measurements as well, to track these changes over time. To determine what markers influence immune reconstruction, we performed multiple linear regression, logistic regression, and PCA.

### 3.4. Linear Regression Approach

For the linear regression approach, measurements of CD4+, CD4+/CD8+ ratio, and the delta of those values were considered.

For CD4+ count reconstruction at the 12‐month endpoint, the only value that had significant statistical influence was the number of CD4+ cells at baseline. For every 1% increase in CD4+ lymphocytes at baseline, the total number of CD4+ cells after 12 months increased by 12.4 cells/μL (95% CI: 1.9040–22.9143; *p* = 0.02).

For CD4+/CD8+ ratio growth, two values were significant. With every 1% of PD‐L1 CD4+ cells at the 12‐month endpoint, the ratio grew by 0.03 (95% CI: 0.005998–0.06446; *p* = 0.02). A similar influence was seen with PD‐1 on CD19+ B‐cells; with every 1% measured at the 12‐month endpoint, the ratio grew by 0.03 (95% CI: 0.008157–0.05230; *p* = 0.009).

Similar dependencies were found when we assessed the delta of CD4+ cells over 12 months, with a significant influence of CD4+ cells at baseline. For every 1% increase in CD4+ lymphocytes at baseline, the delta CD4+ measured after 12 months increased by 10.5 cells/μL (95% CI: 3.5297–17.5050; *p* = 0.04).

In addition to the baseline number of CD4+ cells, PD‐1 and PD‐1L account for very minor changes in immunological reconstruction, mainly affecting the CD4+/CD8+ ratio. This suggests that as the percentage of immune exhaustion receptors increases over the 12‐month observation period in both CD4+ and CD19+ cells, the CD4+/CD8+ ratio also increases. This may be due to global immune recovery, indicating that as immune recovery occurs, more lymphocytes exhibit signs of immune exhaustion.

### 3.5. Logistic Regression Approach

For the logistic regression approach, immune reconstruction was measured for the endpoints listed above: CD4+ lymphocyte counts of 500 and 800 cells/μL and the CD4+/CD8+ ratio, with a value of >0.8 for reconstruction. Logistic regression could not be applied to reconstructions with a value of >1.0.

Regarding the immune reconstruction of CD4+ counts over 500 cells/μL, three of the analyzed variants were found to be significant, as follows:

1. With every 1% increase in PD‐L1 expression on T lymphocytes (CD3+) at baseline, the chances of immune reconstruction within 12 months dropped by 23.7% (*p* = 0.04).
2. For every 1% of CD4+ lymphocyte at baseline, the chance of immune reconstruction in 12 months grew by 7.8% (*p* = 0.003).
3. With every 1% increase in PD‐1 expression on T lymphocytes (CD3+) at the 12‐month checkpoint, the chance of immune reconstruction grew by 3.8% (*p* = 0.03).

For the immune reconstruction of CD4+ count above 800 cells/μL, no statistical significance was found.

In this calculation, we found a strong correlation between the immune exhaustion receptor PD‐L1 on lymphocytes (CD3+) at baseline and significantly lower chances of immune reconstruction. The opposite phenomenon was observed with PD‐1 on lymphocytes (CD3+) at the 12‐month checkpoint, as indicated by a linear regression calculation. This outcome may have been reached due to global immune recovery, suggesting that as immune recovery occurs, more lymphocytes exhibit signs of immune exhaustion.

For the immune reconstruction of CD4+/CD8+ ratio >0.8, only one of the analyzed variants was found to be significant:

1. For every 1% of CD4+ lymphocyte at baseline, the chance of immune reconstruction in 12 months grew by 7.6% (*p* = 0.002).

### 3.6. PCA

We conducted PCA on quantitative variables in the dataset to evaluate any clustering patterns influenced by the categorical variable immune reconstruction (500 cells/μL). The first two principal components (PC1 and PC2) explained 28.4% and 15.2% of the total variance, respectively, accounting for 43.7% of the dataset’s variability.

**Figure 2:**
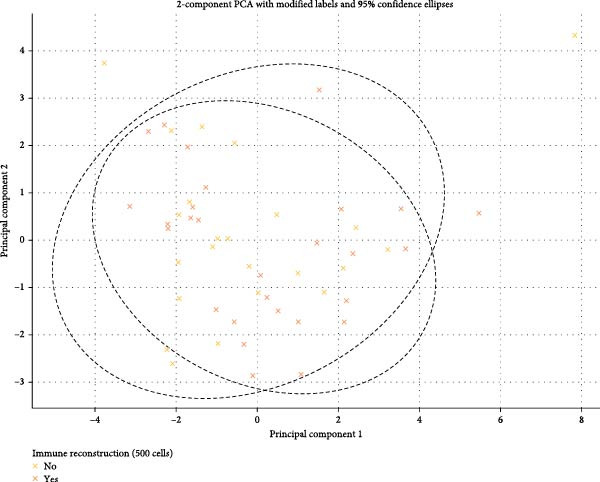
Two‐component PCA for immune reconstruction (500 cells/μL).

To test for potential clustering by immune reconstruction (500 cells/μL) categories, we performed ANOVA tests on the PC1 and PC2 scores. For PC1, the ANOVA test yielded an F‐statistic of 0.374 and a *p*‐value of 0.543. For PC2, the ANOVA test for PC2 scores showed an F‐statistic of 0.449 and a *p*‐value of 0.506. Since the *p*‐values for both PC1 and PC2 are above the 0.05 threshold, no statistically significant differences were found between the means of immune reconstruction (500 cells/μL) groups. This finding suggests that immune reconstruction (500 cells/μL) does not significantly impact clustering in the PCA space for these components. The lack of significant separation implies that the quantitative variables do not differ markedly across immune reconstruction (500 cells/μL) categories within the first two principal components. In short, the immune reconstruction (500 cells/μL) categorical variable does not appear to influence the dataset’s structure in terms of clustering within PC1 and PC2 (Figure 2).

For immune reconstruction to 800 cells/μL of CD4+ T lymphocytes, a similar process was performed.

PCA was conducted on the quantitative variables in the dataset to evaluate any clustering patterns influenced by the categorical variable “immune reconstruction” (800 cells). The first two principal components (PC1 and PC2) explained 21.3% and 14.8% of the total variance, respectively, accounting for 36.1% of the dataset’s variability.

**Figure 3:**
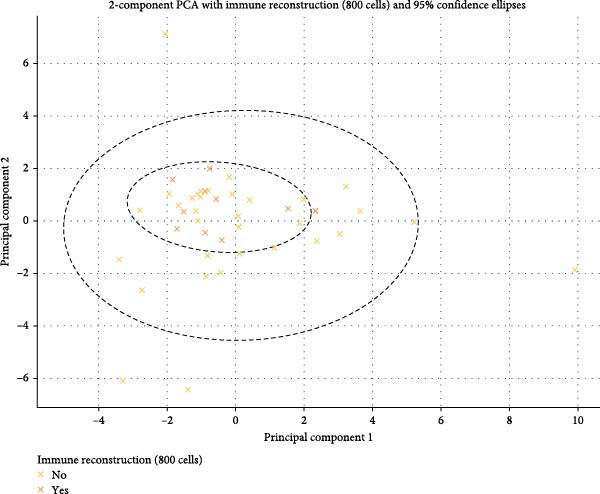
Two‐component PCA for immune reconstruction (800 cells/μL).

To test for potential clustering by immune reconstruction (800 cells/μL) categories, we performed an ANOVA on the PC1 and PC2 scores. For PC1, the ANOVA test yielded an F‐statistic of 0.500 and a *p*‐value of 0.483. For PC2, the ANOVA test showed an F‐statistic of 0.891 and a *p*‐value of 0.351. The *p*‐values for PC1 and PC2 indicate that there are no statistically significant differences between the means of immune reconstruction (800 cells/μL) groups, which, in turn, indicates that immune reconstruction (800 cells/μL) does not significantly impact clustering in the PCA space for these components. Based on this outcome, the quantitative variables do not appear to differ substantially across immune reconstruction (800 cells/μL) categories within the first two principal components. In other words, the immune reconstruction (800 cells/μL) categorical variable did not impact the dataset’s structure regarding clustering in PC1 and PC2 (Figure 3).

## 4. Discussion

### 4.1. Summary of Findings

In this longitudinal study involving 52 people living with HIV undergoing ART, we investigated the role of PD‐1 and its ligand PD‐L1 expressions on immune cells concerning immune reconstitution over 12 months. Our primary findings indicate that PD‐1 and PD‐L1 expressions on immune cells are weakly associated with immune recovery metrics in ART‐treated individuals. Specifically, higher baseline PD‐L1 expression on CD3+ T cells was associated with a reduced likelihood of immune recovery, as the CD4+ T‐cell counts exceeded 500 cells/μL. Conversely, a higher PD‐1 expression on CD3+ T cells at the 12‐month follow‐up was associated with increased odds of achieving this immune recovery threshold. Furthermore, linear regression analyses revealed that increased PD‐L1 expression on CD4+ T cells and PD‐1 expression on CD19+ B cells at the 12‐month endpoint correlated with higher CD4+/CD8+ ratios. Meanwhile, PCA did not demonstrate any significant clustering of immune recovery patterns based on PD‐1/PD‐L1 expression levels and immune cell counts.

### 4.2. Interpretation of PD‐1/PD‐L1’s Associations With Immune Recovery

The observed association between higher baseline PD‐L1 expression on CD3+ T cells and a reduced likelihood of immune recovery suggests that elevated PD‐L1 hinders effective immune reconstitution during ART. PD‐L1, expressed on antigen‐presenting cells and various tissues, interacts with PD‐1 on T cells to attenuate T‐cell activity, contributing to immune exhaustion. Elevated PD‐L1 levels at baseline may reflect a heightened state of immune exhaustion, which could impair the capacity for immune recovery despite effective viral suppression.

Conversely, the positive association between higher PD‐1 expression on CD3+ T cells at the 12‐month follow‐up and the increased odds of immune recovery is intriguing. One possible explanation is that as immune reconstitution progresses, there is a concomitant increase in lymphocyte numbers, including those expressing PD‐1. This pattern could reflect a reactivation or expansion of T‐cell populations that, while expressing PD‐1, still contribute to immune recovery. The upregulation of PD‐1 in this context may represent a regulatory mechanism that prevents the overactivation of the recovering immune system. However, this association should be interpreted with caution, as it may reflect the immunoregulatory role of PD‐1 in tempering immune activation during reconstitution, rather than serving purely as a marker of dysfunction.

The positive correlations between increased PD‐L1 expression on CD4+ T cells and PD‐1 expression on CD19+ B cells at the 12‐month endpoint, along with higher CD4+/CD8+ ratios, further support the notion that complex regulatory changes in immune cell subsets accompany immune recovery. The CD4+/CD8+ ratio is a [26–28]. The observed associations suggest that PD‐1/PD‐L1 expressions play nuanced roles in modulating immune recovery dynamics.

Although certain PD‐1/PD‐L1 expression patterns showed statistically significant associations with an improved CD4+/CD8+ ratio, the overall predictive contribution of these markers to immune reconstitution was limited and inconsistent across analytical methods. These observations indicate that while PD‐1/PD‐L1 may reflect ongoing immune remodeling, their standalone value as biomarkers of immune recovery remains weak.

### 4.3. Comparison With Previous Studies

Previous research has established that elevated PD‐1 expression is associated with immune exhaustion, higher viral loads, and lower CD4+ T‐cell counts in untreated HIV infection [29–31]. Studies have also shown that ART can reduce PD‐1 expression levels over time, indicating that it partially restores immune function [32]. However, the relationship between PD‐1/PD‐L1 expression and immune recovery during ART remains relatively unclear.

Our findings partially contrast with earlier studies suggesting that high PD‐1 expression is uniformly detrimental to immune recovery. The positive association observed in the present study between PD‐1 expression on CD3+ T cells at the 12‐month endpoint and immune recovery may reflect differences in study design, patient populations, or the timing of measurements. During ART‐mediated viral suppression, the role of PD‐1 expression might shift from a marker of immune exhaustion to a regulatory checkpoint that fine‐tunes immune reconstitution.

### 4.4. Possible Explanations of the Findings

Several factors may explain the weak and sometimes paradoxical associations observed between PD‐1/PD‐L1 expressions and immune recovery. The first is dynamic changes during ART. ART leads to rapid viral suppression and gradual immune restoration, and the expressions of PD‐1 and PD‐L1 may fluctuate during this process, reflecting changes in immune activation and regulatory mechanisms. Early in the treatment process, high PD‐L1 expression may impede immune recovery, while increased PD‐1 expression might be part of a controlled immune reconstitution later in the process.

Compensatory mechanisms might also have an impact. The immune system employs various checkpoints to maintain homeostasis. As immune cells recover, the upregulation of inhibitory receptors, such as PD‐1, could prevent hyperactivation and potential autoimmunity. This could explain the positive association between PD‐1 expression at 12 months and immune recovery.

The heterogeneity of immune responses is another possible factor affecting the observed associations between PD‐1/PD‐L1 expressions and immune recovery. Individual variability in immune responses to HIV and ART may influence PD‐1/PD‐L1 expressions and their impact on immune recovery. Genetic factors, coinfections, and differences in immune system baseline status could contribute to the observed associations.

Finally, measurement limitations could have played a role. Flow cytometry analyses provide snapshots of PD‐1/PD‐L1 expressions but may not capture functional aspects of immune cells. However, the mere presence of PD‐1 does not necessarily indicate functional exhaustion, especially in the context of ART and immune reconstitution.

### 4.5. Limitations of the Study

Our study has several limitations that should be acknowledged First, the small sample size of 52 participants means the study may have been unable to detect subtle associations. Furthermore, it is difficult to generalize findings to the broader HIV‐infected population. Larger studies are needed to validate the observations made in the current study.

The short follow‐up period may also be a limitation, as a 12‐month follow‐up may not be sufficient to observe long‐term patterns of immune recovery and changes in PD‐1/PD‐L1 expression. Extended longitudinal studies could provide more comprehensive insights.

Furthermore, the present study could be limited in terms of HIV subtype variability. While we included patients with different HIV subtypes, the small sizes of these groups did not allow us to conduct robust subtype‐specific analyses, and subtype‐related differences in immune responses could affect the generalizability of our results.

This article presents preliminary data on a relatively small group to fully assess the impact of PD‐1 on immune reconstitution in people living with HIV. However, our dataset lacks functional validation (e.g., cytokine profiling, proliferation assays) and does not include high‐resolution longitudinal tracking of PD‐1 expression dynamics, which limits the mechanistic interpretation of our findings. Additionally, while PCA was applied to evaluate the overall structure and variability, alternative nonlinear dimensionality reduction methods, such as UMAP or clustering algorithms, may offer deeper insights into complex immune phenotypes in future analyses.

## 5. Conclusion

We found that PD‐1 and PD‐L1 expressions on immune cells are weakly associated with immune recovery metrics in HIV‐infected individuals undergoing ART. Our findings indicate that higher baseline PD‐L1 expression on CD3+ T cells may impede immune reconstitution, while increased PD‐1 expression on CD3+ T cells at 12 months may reflect ongoing regulatory processes during immune recovery. These findings highlight the complex role of the PD‐1/PD‐L1 pathway in HIV‐associated immune reconstitution. Further research is necessary to elucidate the mechanisms involved and to explore potential therapeutic strategies intended to optimize immune recovery in this population.

## Ethics Statement

The study protocol was approved by the Bioethical Committee of the Pomeranian Medical University in Szczecin (Approval number KB‐006/40/2023).

## Conflicts of Interest

The authors declare no conflicts of interest.

## Author Contributions

**Bogusz Aksak-Wąs**: conceptualization, funding acquisition, investigation, methodology, supervision, validation, visualization, writing – original draft. **Karolina Skonieczna-Żydecka**: formal analysis, investigation, methodology, supervision, validation, visualization, writing – original draft. **Miłosz Parczewski**: conceptualization, methodology, supervision, validation, visualization. **Rafał Hrynkiewicz**: data curation, resources, methodology. **Filip Lewandowski**: data curation, resources, methodology. **Karol Serwin**: data curation, resources, methodology. **Kaja Mielczak**: data curation, resources, methodology. **Adam Majchrzak**: investigation, resources, methodology. **Franciszek Lenkiewicz**: investigation, resources, methodology. **Paulina Niedźwiedzka-Rystwej**: conceptualization, data curation, investigation, supervision, validation, visualization, writing – original draft and revision.

## Funding

The study was funded by the Polish National Science Centre (NCN), MINIATURA 7 (Grant DEC‐2023/07/X/NZ6/00001); Polish AIDS Society (PTN AIDS): The influence of immune depletion receptors PD‐1 and PD‐1 ligand on the immune reconstitution of PLWH; Minister of Science under the “Regional Excellence Initiative” Program for 2024‐2027 (Grant RID/SP/0045/2024/01).

## Supporting Information

Additional supporting information can be found online in the Supporting Information section.

## Supporting information


**Supporting Information** Table S1: Summary statistics were provided for baseline and 12‐month checkpoint analysis.

## Data Availability

Because the publication process for all associated articles is still in progress, raw data are available upon request from the authors. To request data, please contact the corresponding author: karolina.skonieczna.zydecka@pum.edu.pl.
