# Supplementary material for: Influence of PD‐1 and PD‐1L Immune Exhaustion Receptors on Immune Reconstruction in People Living With HIV
Source: J Immunol Res. 2025 Sep 30;2025:2462382. doi: 10.1155/jimr/2462382 (PMC12481824; doi:10.1155/jimr/2462382)
Supplement: Supplementary file 1 — Supporting Information Table S1: Summary statistics were provided for baseline and 12‐month checkpoint analysis. [file JIMR-2025-2462382-s001.docx]

Suplementary table 1: Summary statistics table.

|  | N | Minimum | Maximum | Mean | Median | SD | 25 - 75 P |
| --- | --- | --- | --- | --- | --- | --- | --- |
| **Baseline measurement** | | | | | | | |
| Frequency of lymphocytes T(CD3+) [%] | 52 | 0,1 | 78,4 | 26,125 | 22,1 | 19,6309 | 9,450 to 36,500 |
| Frequency of lymphocytes T(CD3+) PD-1 [%] | 52 | 11,1 | 69,6 | 29,635 | 27,3 | 11,225 | 21,100 to 36,400 |
| Frequency of lymphocytes T(CD3+) PD-L1 [%] | 52 | 0 | 16,7 | 3,075 | 0,8 | 4,0636 | 0,200 to 5,000 |
| Frequency of lymphocytes Th(CD4+) [%] | 52 | 7,9 | 75,7 | 43,625 | 46,7 | 17,9644 | 28,200 to 56,150 |
| Frequency of lymphocytes Th(CD4+) PD-1 [%] | 52 | 0 | 61 | 26,444 | 24,4 | 12,3713 | 18,950 to 32,700 |
| Frequency of lymphocytes Th(CD4+) PD-L1 [%] | 52 | 0 | 16,1 | 2,765 | 0,45 | 4,1192 | 0,0500 to 5,300 |
| Frequency of lymphocytes B(CD19+) [%] | 52 | 0,1 | 45,4 | 12,46 | 12,05 | 7,5607 | 6,650 to 17,000 |
| Frequency of lymphocytes B(CD19+) PD-1 [%] | 52 | 0 | 5,5 | 0,94 | 0,6 | 1,1005 | 0,300 to 1,200 |
| Frequency of lymphocytes B (CD19+) PD-L1 [%] | 52 | 0 | 4,9 | 0,515 | 0,2 | 0,9851 | 0,000 to 0,600 |
| **Measurements in 12-month checkpoint** | | | | | | | |
| Frequency of lymphocytes T(CD3+) [%] | 52 | -7,5 | 76 | 21,598 | 18,5 | 20,2553 | 4,300 to 32,150 |
| Frequency of lymphocytes T(CD3+) PD-1 [%] | 52 | -73 | 46,9 | 4,54 | 11,55 | 25,6032 | -9,350 to 20,450 |
| Frequency of lymphocytes T(CD3+) PD-L1 [%] | 52 | -100 | 16,7 | -3,231 | 0,4 | 19,2008 | -1,850 to 4,150 |
| Frequency of lymphocytes Th(CD4+) [%] | 52 | -18,1 | 75,7 | 29,085 | 30,45 | 22,0058 | 14,400 to 43,100 |
| Frequency of lymphocytes Th(CD4+) PD-1 [%] | 52 | -75,8 | 61 | 11,306 | 12,45 | 24,1544 | 0,000 to 27,350 |
| Frequency of lymphocytes Th(CD4+) PD-L1 [%] | 52 | -6,5 | 16,1 | 2,198 | 0,4 | 4,1978 | 0,000 to 3,100 |
| Frequency of lymphocytes B(CD19+) [%] | 52 | -18 | 45 | 6,6 | 5,95 | 9,6531 | 0,500 to 11,900 |
| Frequency of lymphocytes B(CD19+) PD-1 [%] | 52 | -62,2 | 4,5 | -2,187 | 0,3 | 9,428 | -0,400 to 0,600 |
| Frequency of lymphocytes B (CD19+) PD-L1 [%] | 52 | -66,5 | 4,8 | -2,098 | 0 | 9,7088 | -1,000 to 0,300 |
